# Plasma‐based proteomic analysis identifies distinct molecular subtypes of Alzheimer's disease pathogenesis

**DOI:** 10.1002/alz70855_106114

**Published:** 2025-12-24

**Authors:** Hanjun Zhao, Liu Shi, Rashmi Maurya, Shisong Jiang, Alejo J Nevado‐Holgado, Laura M Winchester, EMIF‐AD Consortia

**Affiliations:** ^1^ Department of Psychiatry, University of Oxford, Oxford, United Kingdom; ^2^ Department of Oncology, University of Oxford, Oxford, United Kingdom

## Abstract

**Background:**

Alzheimer's disease (AD) is characterized by significant molecular heterogeneity. Plasma proteomics offers an opportunity to identify novel biomarkers and understand disease mechanisms. Further classification by amyloid and tau profile allows us to focus on distinct AD pathophysiology.

**Method:**

We analyzed 3,635 plasma proteins using SOMAscan assay platform from 973 participants in European Medical Information Framework (EMIF)‐AD Multimodal Biomarker Discovery (MBD) study. By integrating CSF amyloid β (A) and phosphorylated tau (T) profiles, we performed linear regression analysis to identify differentially abundant proteins (DAPs) in A+T+ patients (*n* =  335) versus A‐T‐ controls (*n* =  297) and applied LightGBM to determine proteins that accurately distinguish biologically defined AD. To characterize AD heterogeneity, we applied non‐negative matrix factorization (NMF) to A+T+ patients’ DAPs, revealing distinct molecular subtypes with varying clinical trajectories.

**Result:**

We identified 11 core DAPs associated with distinct A/T categories across the AD spectrum, along with 10 early DAPs only in A+T‐ and 190 late DAPs only in A+T+. LRRN1 emerged as the most robust predictor of A+T+ defined AD (AUC = 0.757). Using 201 DAPs in A+T+ patients, we classified biologically defined AD into four distinct molecular subtypes, each enriched in different biological pathways, including complement activation, lipid storage regulation, glucose metabolism, and cytokine production. Notably, the subtype 4, characterized by elevated LRRN1 expression, exhibited the strongest association with cognitive deterioration.

**Conclusion:**

This study identifies protein signatures associated with amyloid β and tau pathology in real‐world clinical cohorts, highlighting LRRN1 as both a promising candidate diagnostic biomarker and potential therapeutic target for specific AD subtypes.

